# Prognostic implication of venoarterial extracorporeal membrane oxygenation in acute myocardial infarction-related cardiogenic shock

**DOI:** 10.1186/s40560-025-00807-w

**Published:** 2025-07-02

**Authors:** Jonghu Shin, Eun-Mi Kang, Sang-Hyup Lee, Minju Heo, Yong-Joon Lee, Seung-Jun Lee, Sung-Jin Hong, Jung-Sun Kim, Byeong-Keuk Kim, Young-Guk Ko, Donghoon Choi, Myeong-Ki Hong, Yangsoo Jang, Chul-Min Ahn

**Affiliations:** 1 Division of Cardiology, Severance Hospital, Yonsei University College of Medicine, 50-1 Yonsei-Ro, Seodaemun-Gu, Seoul, 03722 South Korea; 2 Division of Cardiovascular Surgery, Severance Cardiovascular Hospital, Yonsei University College of Medicine, Seoul, South Korea; 3 Research Support Department, Yonsei Biomedical Research Center, Yonsei University College of Medicine, Seoul, South Korea; 4 Division of Cardiology, CHA University College of Medicine, Seongnam, South Korea

**Keywords:** Extracorporeal membrane oxygenation, Cardiogenic shock, Cardiac arrest, Lactate

## Abstract

**Background:**

Given the conflicting results regarding the clinical outcomes of venoarterial extracorporeal membrane oxygenation (VA-ECMO) based on etiology, its benefit for patients with cardiogenic shock (CS) remains controversial. This study aimed to report the real-world clinical outcomes of VA-ECMO treatment for patients with CS, based on the presence of acute myocardial infarction (AMI).

**Methods:**

Patients treated with peripheral VA-ECMO between 2008 and 2023 at a tertiary cardiovascular center were included and classified into two groups based on CS etiology (AMI-CS and non-AMI-CS). Logistic regression models were used to compare in-hospital mortality and to identify prognostic predictors.

**Results:**

Among the 667 patients included, 264 (39.6%) were classified as having AMI-CS. The rate of cardiac arrest before VA-ECMO initiation was higher in the AMI-CS group than in the non-AMI-CS group (69.7% vs. 55.8%; *P* < 0.001). Patients in the AMI-CS group were older (66 vs. 61 years; *P* < 0.001), more likely to be male (82.6% vs. 57.3%; *P* < 0.001), and had a lower left ventricular (LV) ejection fraction (20% vs. 25%; *P* < 0.001) than those in the non-AMI-CS group. The AMI-CS group had a lower in-hospital mortality rate (58.6% vs. 69.7%; odds ratio, 0.46; 95% confidence interval, 0.29–0.75; *P* = 0.002) compared with the non-AMI-CS group. The independent predictors of favorable clinical outcomes after VA-ECMO included younger age, shorter cardiac arrest duration, absence of severe LV dysfunction, absence of renal replacement therapy, higher hemoglobin levels, higher arterial pH, and lower lactate levels. The association between in-hospital mortality and AMI-CS was also demonstrated in the propensity score matching analysis.

**Conclusions:**

In this single-center study, AMI-CS was associated with a lower in-hospital mortality than non-AMI-CS after VA-ECMO treatment.

**Graphical Abstract:**

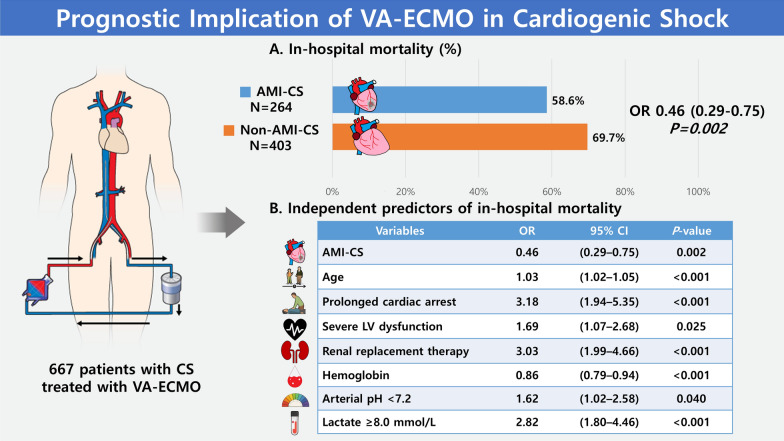

**Supplementary Information:**

The online version contains supplementary material available at 10.1186/s40560-025-00807-w.

## Introduction

Cardiogenic shock (CS) is a highly fatal condition caused by organ hypoperfusion due to cardiac dysfunction [1]. Despite advancements in critical and cardiovascular care, the mortality rate remains high in patients with CS, which is evident not only in registry data, but also in randomized clinical trials [2–4].

Venoarterial extracorporeal membrane oxygenation (VA-ECMO) is a potent mechanical circulatory support (MCS) device capable of rapidly restoring sufficient organ perfusion in patients with CS [5, 6]. Notably, VA-ECMO can be applied immediately, providing hemodynamic stabilization even in patients with biventricular failure and during resuscitation [5, 6]. However, VA-ECMO use remains contentious owing to the lack of evidence on its efficacy and potential for fatal complications. Consequently, contemporary guidelines for the management of CS and acute heart failure (HF) recommend the selective use of VA-ECMO, mainly based on the theoretical background [7, 8].

Acute myocardial infarction (AMI) ranks among the leading etiologies of CS [9]. Although the in-hospital mortality of patients with AMI-CS seems higher than that of patients with non-AMI-CS**,** [9] several observational studies have shown conflicting results regarding the prognostic difference after VA-ECMO treatment between patients with AMI-CS and non-AMI-CS [10, 11]. Moreover, recent randomized trials did not elucidate the benefit of VA-ECMO in patients with AMI-CS [12, 13]. Hence, this study aimed to evaluate the effect of VA-ECMO and compare the clinical outcomes between patients with AMI-CS and non-AMI-CS using real-world registry data.

## Methods

### Data collection and study populations

Patients treated with peripheral ECMO for CS between 2008 and 2023 at a tertiary cardiovascular hospital were retrospectively included. CS was defined as a cardiac disorder that results in a systolic blood pressure < 90 mmHg for ≥ 30 min or the need for vasopressors, inotropes, or MCS to maintain systolic blood pressure ≥ 90 mmHg with evidence of tissue hypoperfusion [14]. The major exclusion criteria included: 1) age < 18 years; 2) venovenous ECMO initiation; and 3) post-cardiotomy CS, which was defined as CS in the perioperative period, with low cardiac output syndrome without specific alternative causes [14].

The included patients were classified into two groups based on CS etiology (AMI-CS and non-AMI-CS groups). AMI-CS was defined as CS resulting from AMI or AMI-related complications. AMI was defined as acute myocardial injury with clinical evidence in accordance with the fourth universal definition of myocardial infarction [15]. Patients without suspicious culprit coronary lesions were classified into the non-AMI-CS group, which included HF-CS, secondary CS, or mixed CS, according to the Shock Academic Research Consortium classification [14].

Clinical and laboratory information obtained closest to, but prior to, ECMO initiation was retrospectively collected. Echocardiographic data prior to ECMO initiation were collected based on available recordings. In cases where such data were unavailable due to urgent clinical circumstances, echocardiographic findings within 24 h after ECMO initiation were used instead. The Society for Cardiovascular Angiography and Interventions and Cardiogenic Shock Working Group (SCAI-CSWG) stage was estimated based on previous literature [1].

### Patient management and study outcome

All data regarding patient management and study outcomes were reviewed based on standard clinical practice by the study protocol.

VA-ECMO was initiated at the discretion of the physicians and was based on unresponsiveness to inotropes and vasopressors after correction of hypovolemia or prolonged cardiac arrest. During VA-ECMO, intravenous heparin was administered to maintain an activated clotting time (ACT) of 180–200 s, with target ACT values adjusted by physicians according to the patient’s thrombotic and bleeding risks. Distal perfusion catheter insertion was recommended, and left ventricle (LV) unloading was considered when indicated by LV distention or pulmonary congestion, based on physician judgement [16]. Weaning from VA-ECMO was undertaken when physicians assessed the patient as sufficiently recovered to survive without VA-ECMO support. On the other hand, VA-ECMO was removed on the premise of death when recovery was deemed improbable by physicians and legal representatives. The primary outcome of this study was in-hospital mortality after the initiation of VA-ECMO.

### Statistical analyses

Variables are presented as mean ± standard deviation, median and interquartile range (IQR), or frequency and percentage, as appropriate. Student’s t-test, Mann–Whitney U-test, chi-square test, or Fisher’s exact test were used to compare variables, as appropriate. Potential monotonic trends in the time-series data were investigated using linear regression analysis. A density plot was generated to visualize the proportion of patients dying at a certain time point after the initiation of VA-ECMO to specify when the greatest number of deaths occurred.

Logistic regression models were constructed to explore factors affecting in-hospital mortality following VA-ECMO initiation. Odds ratios (ORs) and 95% confidence intervals (CIs) were calculated using logistic regression. As patients who died within 24 h of VA-ECMO initiation were considered to have futile implementation of VA-ECMO, a sensitivity analysis was conducted by performing the same analysis in the population excluding patients who died within 24 h. Additionally, given the unique clinical characteristics associated with extracorporeal cardiopulmonary resuscitation (ECPR), a separate sensitivity analysis excluding ECPR patients was also performed.

Furthermore, propensity score matching was performed as an additional analysis to adjust for potential baseline differences between the AMI-CS and non-AMI-CS groups. Nearest-neighbor matching without replacement was applied using a 1:1 ratio and a caliper width of 0.2 times the standard deviation of the logit of the propensity score. The propensity score model included the following variables: age, sex, ECPR, LV ejection fraction, LV unloading, use of renal replacement therapy, hemoglobin level, platelet count, pH, lactate level, and SCAI-CSWG shock stage. The same logistic regression analysis was repeated in the matched cohort.

All analyses were two-sided, and *P*-values < 0.05 were considered significant. R software, version 4.4.1 (R Core Team) was used for the analysis.

## Results

### Baseline characteristics

**Fig. 1:**
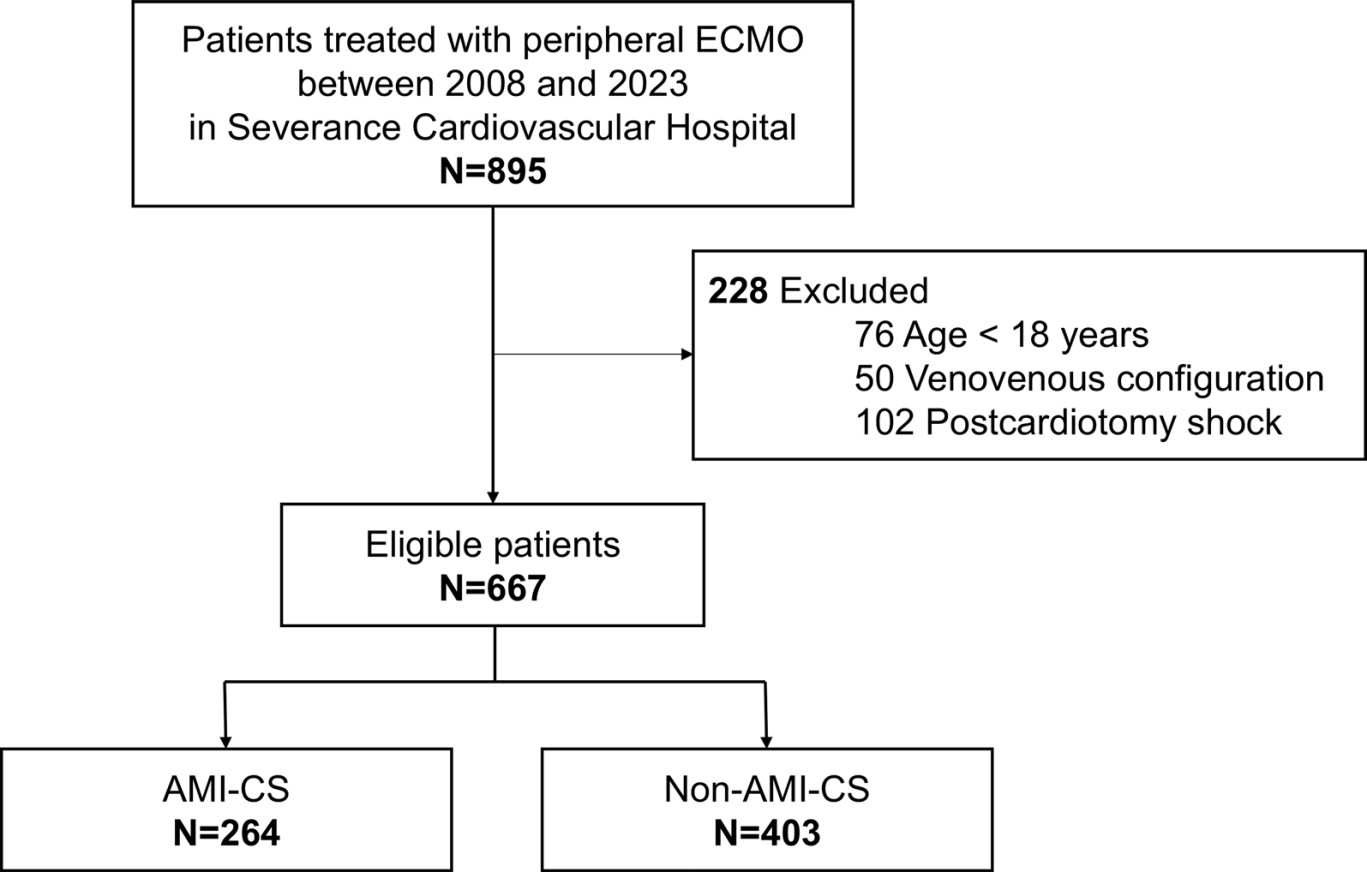
Flowchart of patient inclusion in this study. Patients with CS treated with peripheral VA-ECMO were included. The included patients were classified into the AMI-CS and non-AMI-CS groups based on the etiology of CS. AMI, acute myocardial infarction; CS, cardiogenic shock; VA-ECMO, venoarterial extracorporeal membrane oxygenation

A total of 895 patients treated with peripheral ECMO between 2008 and 2023 were screened, of whom 667 were finally included in this study (Fig. 1). The baseline characteristics of the patients are presented in Table 1. Overall, the median age was 63 (IQR, 51–72) years and 449 (67.3%) patients were male. Before VA-ECMO initiation, 409 (61.3%) patients experienced cardiac arrest. ECPR was performed in 266 (39.9%) patients and 541 (81.1%) patients were in SCAI-CSWG stage E when ECMO was initiated. The median serum lactate level was 10.3 (IQR, 4.9–16.4) mmol/L and the proportion of lactate level ≥ 8.0 mmol/L was 59.1%.

**Table 1:** Baseline characteristics of the included patients

Variables	AMI-CS group (N = 264)	Non-AMI-CS group (N = 403)	Total (N = 667)	*P*-value
Age, years	66 (58–74)	61 (45–72)	63 (51–72)	< 0.001
Male	218 (82.6)	231 (57.3)	449 (67.3)	< 0.001
ECPR	119 (45.1)	147 (36.5)	266 (39.9)	0.033
Cardiac arrest	184 (69.7)	225 (55.8)	409 (61.3)	< 0.001
CPR time (min)	24 (14–44)	28 (15–43)	26 (14–43)	0.369
CPR rhythm				< 0.001
Shockable	75 (40.8)	48 (21.3)	123 (30.1)	
Non-shockable	89 (48.4)	158 (70.2)	247 (60.4)	
Unknown	20 (10.9)	19 (8.4)	39 (9.5)	
LV ejection fraction (%)	20 (13–31)	25 (15–52)	23 (14–41)	< 0.001
SCAI-CSWG shock stage				0.091
Stage C	15 (5.7)	25 (6.2)	40 (6.0)	
Stage D	25 (9.5)	61 (15.1)	86 (12.9)	
Stage E	224 (84.9)	317 (78.7)	541 (81.1)	
Renal replacement therapy	131 (49.6)	241 (60.0)	372 (55.9)	0.011
LV unloading	6 (2.3)	29 (7.2)	35 (5.2)	0.009
HT or LVAD	7 (2.7)	56 (13.9)	63 (9.4)	< 0.001
Hemoglobin, g/dL	12.1 (10.1–14.4)	10.2 (8.6–12.6)	11.2 (9.0–13.3)	< 0.001
Platelet count,/μL	193,000 (140,000–255,500)	135,000 (76,000–203,000)	160,000 (96,500–233,000)	< 0.001
AST, IU/L	82 (33–280)	101 (36–302)	93 (34–287)	0.213
ALT, IU/L	60 (27–167)	65 (25–243)	62 (26–213)	0.483
Creatinine, mg/dL	1.33 (1.06–1.71)	1.34 (1.00–2.01)	1.33 (1.01–1.88)	0.845
pH	7.20 (7.06–7.30)	7.22 (7.10–7.33)	7.21 (7.08–7.32)	0.020
Lactate				
Value, mmol/L	10.6 (5.8–15.1)	10.1 (4.2–17.3)	10.3 (4.9–16.4)	0.535
≥ 8.0 mmol/L	145 (63.9)	198 (56.1)	343 (59.1)	0.076
Length of hospital stay, days	12 (2–26)	10 (1–33)	11 (1–29)	0.645
CPC at discharge				0.478
1–2	40 (38.1)	43 (36.4)	83 (37.2)	
3	61 (58.1)	66 (55.9)	127 (57.0)	
4–5	4 (3.8)	9 (7.6)	13 (5.8)	

Data are presented as the median (interquartile range) or number (%). AMI-CS, acute myocardial infarction-cardiogenic shock; ALT, alanine aminotransferase; AST, aspartate aminotransferase; CPC, cerebral performance category; CPR, cardiopulmonary resuscitation; ECPR, extracorporeal cardiopulmonary resuscitation; HT, heart transplantation; IU, international unit; LV, left ventricle; LVAD, left ventricle assist device; SCAI-CSWG, Society for Cardiovascular Angiography and Interventions and Cardiogenic Shock Working Group

The AMI-CS group comprised 264 (39.6%) patients. Patients in the AMI-CS group were older (66 vs. 61 years; *P* < 0.001), more likely to be male (82.6% vs. 57.3%; *P* < 0.001), more likely to experience cardiac arrest before VA-ECMO (69.7% vs. 55.8%; *P* < 0.001), had a lower LV ejection fraction (20% vs. 25%; *P* < 0.001), and had higher hemoglobin levels (12.1 vs. 10.2 g/dL; *P* < 0.001) and platelet counts (193,000 vs. 135,000/μL; *P* < 0.001), compared with patients in the non-AMI-CS group. Although the initial arterial pH was lower in the AMI-CS group (7.20 vs. 7.22, *P* = 0.020), the proportion of lactate level ≥ 8.0 mmol/L (63.9% vs. 56.1%; *P* = 0.076) and the absolute value (10.6 vs. 10.1 mmol/L; *P* = 0.535) showed no significant difference between the two groups. The length of hospital stay did not differ between the two groups (12 vs. 10 days; *P* = 0.645). Long-term cardiac replacement therapy, including heart transplantation (HT) and left ventricular assist device (LVAD), was performed in 7 (2.7%) patients in the AMI-CS group and 56 (13.9%) patients in the non-AMI-CS group (*P* < 0.001). Renal replacement therapy (49.6% vs. 60.0%; *P* = 0.011) and LV unloading (2.3% vs. 7.2%; *P* = 0.009) were performed more frequently in the non-AMI-CS group than the AMI-CS group.

The lesions and procedural characteristics of patients with AMI-CS are described in Supplemental Table S1. ST-elevation MI accounted for 66.7% of the patient diagnoses, and 49.6% of the patients had three-vessel disease. The most common culprit vessel was the left anterior descending artery (41.9%). The overall procedural success rate was 78.7%.

Among the patients included in the non-AMI-CS group, 126 (31.3%) had acute-on-chronic HF-CS with known cardiomyopathies, such as dilated cardiomyopathy, ischemic cardiomyopathy, or hypertrophic cardiomyopathy (Supplemental Figure S1). In contrast, 56 (13.9%) patients had de novo HF-CS, including acute myocarditis, and 221 (54.8%) patients had secondary or mixed CS.

### Time-to-death analysis

The density plot for time-to-death data showed that patients treated with VA-ECMO most frequently died approximately 24 h after VA-ECMO initiation, followed by a plateau observed approximately 7 days later (Supplemental Figure S2). In the first 30 days after VA-ECMO initiation, the overall mortality rates were 55.7% in AMI-CS patients and 62.0% in non-AMI-CS patients, respectively. Baseline characteristics stratified by initiation-to-death interval are detailed in Supplemental Table S2. Among the 442 patients who died during the index hospitalization, 145 (32.8%) died within 24 h of VA-ECMO initiation. Patients who died within 24 h were older (70 vs. 65 vs. 59 years; *P* < 0.001) and more likely to experience cardiac arrest (80.0% vs. 59.3% vs. 52.0%; *P* < 0.001) than those who died after 24 h or were discharged alive. They also had lower arterial pH (7.14 vs. 7.19 vs. 7.27; *P* < 0.001) along with higher lactate levels (14.0 vs. 12.2 vs. 6.3 mmol/L; *P* < 0.001), despite higher LV ejection fractions (46% vs. 19% vs. 23%; *P* < 0.001), respectively. Of note, 43 (19.1%) of the 225 patients who were discharged alive received HT or LVAD as cardiac replacement therapy.

The proportion of AMI-CS patients was lower among those who died within 24 h (35.2% vs. 36.4% vs. 46.7%; *P* = 0.027), whereas the proportion of mixed or secondary CS was higher in this group (48.3% vs. 33.7% vs. 22.7%; *P* < 0.001) than in the other groups (Supplemental Figure S3).

### Clinical outcome of in-hospital mortality

**Fig. 2:**
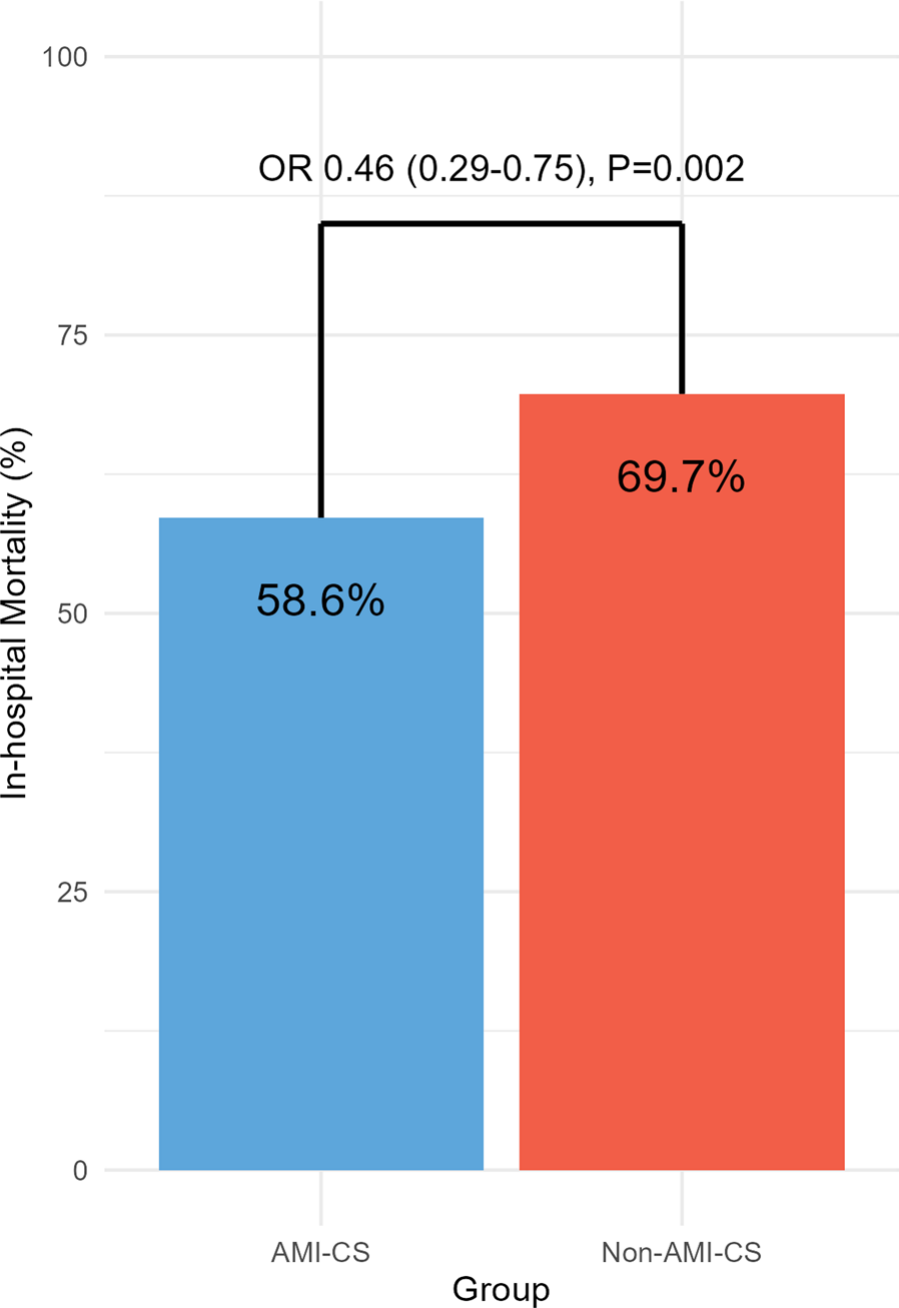
In-hospital mortality according to CS etiology. The bar graph compares in-hospital mortality rates between the AMI-CS and non-AMI-CS groups. The *P*-value was calculated using a multivariate logistic regression analysis. AMI, acute myocardial infarction; CI, confidence interval; CS, cardiogenic shock; OR, odds ratio

**Fig. 3:**
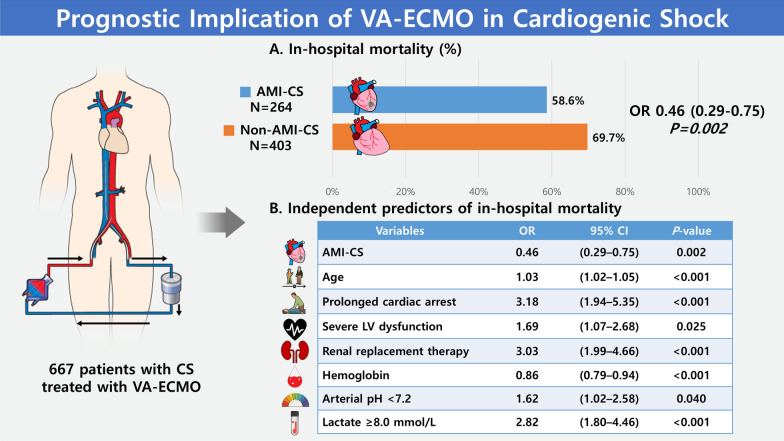
Prognostic Implication of VA-ECMO in cardiogenic shock. This study compares mortality in patients treated with VA-ECMO for CS, with and without AMI. Patients with AMI were associated with lower in-hospital mortality. Higher hemoglobin level was associated with favorable outcomes, whereas old age, prolonged cardiac arrest, severe LV dysfunction, renal replacement therapy, arterial pH < 7.2, and lactate levels over 8.0 mmol/L were associated with unfavorable outcomes. AMI, acute myocardial infarction; CS, cardiogenic shock; LV, left ventricle; VA-ECMO, venoarterial extracorporeal membrane oxygenation

The overall in-hospital mortality rate was 66.3%. The rate of in-hospital mortality was lower in the AMI-CS group (58.6% vs. 69.7%; OR, 0.46; 95% CI, 0.29–0.75; *P* = 0.002) than in the non-AMI-CS group (Fig. 2). Furthermore, seven independent predictors of in-hospital mortality after VA-ECMO were identified using multivariate regression analysis (Fig. 3). Higher hemoglobin level (OR, 0.86; 95% CI, 0.79–0.94; *P* < 0.001) was associated with favorable clinical outcomes, whereas old age (OR, 1.03; 95% CI, 1.02–1.05; *P* < 0.001), prolonged cardiac arrest (OR, 3.18; 95% CI, 1.94–5.35; *P* < 0.001), severe LV dysfunction (OR, 1.69; 95% CI, 1.07–2.68; *P* = 0.025), renal replacement therapy (OR, 3.03; 95% CI, 1.99–4.66; *P* < 0.001), arterial pH < 7.2 (OR, 1.62; 95% CI, 1.02–2.58; *P* = 0.040), and lactate ≥ 8.0 mmol/L (OR, 2.82; 95% CI, 1.80–4.46; *P* < 0.001) were associated with unfavorable clinical outcomes (Table 2). The rates of in-hospital mortality were lower in AMI-CS patients than in non-AMI-CS patients in sensitivity analyses excluding patients who died within 24 h (OR, 0.50; 95% CI, 0.30–0.85; *P* = 0.011) (Supplemental Table S3) and excluding ECPR patients (OR, 0.49; 95% CI, 0.26–0.91; *P* = 0.025) (Supplemental Table S4).

**Table 2:** Logistic regression models for in-hospital mortality

Variables	Univariate model		Multivariate model	
	OR (95% CI)	*P*-value	OR (95% CI)	*P*-value
AMI-CS patients	0.61 (0.43–0.87)	0.006	0.46 (0.29–0.75)	0.002
Age	1.02 (1.01–1.04)	< 0.001	1.03 (1.02–1.05)	< 0.001
Male	0.79 (0.55–1.14)	0.211	1.04 (0.66–1.63)	0.879
Prolonged cardiac arrest^a^	2.58 (1.69–4.00)	< 0.001	3.18 (1.94–5.35)	< 0.001
Severe LV dysfunction^b^	1.23 (0.86–1.76)	0.253	1.69 (1.07–2.68)	0.025
Renal replacement therapy	3.35 (2.35–4.79)	< 0.001	3.03 (1.99–4.66)	< 0.001
Left ventricle unloading	0.89 (0.44–1.85)	0.743	–	–
Hemoglobin	0.83 (0.78–0.88)	< 0.001	0.86 (0.79–0.94)	< 0.001
Platelet count per 1000/μL	0.04 (0.01–0.20)	< 0.001	0.26 (0.03–2.42)	0.233
AST per 100 IU/L	1.02 (1.01–1.05)	0.024	1.01 (0.98–1.04)	0.513
ALT per 100 IU/L	1.02 (1.00–1.05)	0.063	0.99 (0.96–1.04)	0.760
Creatinine	1.25 (1.07–1.48)	0.007	0.99 (0.84–1.20)	0.924
pH < 7.2	2.40 (1.69–3.43)	< 0.001	1.62 (1.02–2.58)	0.040
Lactate ≥ 8.0 mmol/L	3.81 (2.67–5.47)	< 0.001	2.82 (1.80–4.46)	< 0.001

AMI-CS, acute myocardial infarction-cardiogenic shock; ALT, alanine aminotransferase; AST, aspartate aminotransferase; CI, confidence interval; IU, international unit; LV, left ventricle; OR, odds ratio

^a^Prolonged cardiac arrest indicates an arrest of duration > 30 min

^b^Severe LV dysfunction indicates an LV ejection fraction < 30%

For a sensitivity analysis using propensity score matching, a total of 294 patients were matched in a 1:1 ratio. In the matched population, baseline characteristics were not different across variables between the two matched groups (Supplemental Table S5). The rate of in-hospital mortality was significantly lower in AMI-CS patients than in non-AMI-CS patients (OR, 0.33; 95% CI, 0.18–0.62; *P* < *0.001*) in the matched population. Independent predictors of in-hospital mortality remained consistent with those identified prior to matching (Supplemental Table S6).

### Time-series analysis

The total volume (*P* for trend < 0.001) and running time (*P* for trend < 0.001) of VA-ECMO increased over time (Supplemental Figure S4). While the overall in-hospital mortality showed a decreasing trend over time (*P* for trend = 0.058), this trend was more prominent in the AMI-CS group (*P* for trend = 0.027) than in the non-AMI-CS group (*P* for trend = 0.181) (Supplemental Figure S5).

## Discussion

The current study demonstrated that AMI-CS was associated with favorable clinical outcomes after VA-ECMO treatment compared to non-AMI-CS. Furthermore, a decreasing trend in in-hospital mortality was observed in AMI-CS patients after VA-ECMO treatment.

The use of VA-ECMO steadily increased over time, which is consistent with the findings of previous studies [17, 18]. VA-ECMO is a crucial strategy not only for critically ill patients at risk of mortality but also for those who are expected to develop rapidly deteriorating CS [1, 5, 6]. In the present study, the total volume of VA-ECMO showed threefold increase in the last 5 years (2019–2023) compared with that in the earliest 5 years (2008–2012), with a corresponding increase in overall running time. This result can be attributed to recent advancements in ECMO technology, including biocompatible membranes, cannulation strategies, use of distal perfusion cannulas, and anticoagulation monitoring systems, leading to favorable clinical outcomes in patients treated with VA-ECMO [19]. Furthermore, with the emergence of exit strategies such as HT or LVAD, patients whose heart function is not expected to recover also have available options [5, 20]. Regarding these advances, the present study showed gradual improvement in in-hospital mortality.

### Randomized trials and real-world data

The overall in-hospital mortality rate of this study population, which was 66.3%, was higher than that reported in the recent statistical document from the extracorporeal life support organization (ELSO) registry [4]. Conversely, randomized trials regarding the impact of VA-ECMO have reported 30-day mortality rates less than 50% [12, 13, 21]. This numerical gap in mortality rates between observational and randomized studies may stem from the differences in the clinical characteristics of the study population [22]. The median lactate level was 10.3 mmol/L in this study, which was higher than that in the randomized trials, which ranged 5.0–8.1 mmol/L [12, 13, 21]. Regarding the strong correlation of lactate level with mortality [23], the higher lactate levels in this study may explain the higher mortality rate compared with previous randomized trials. Nevertheless, the present observational study may offer a more accurate reflection of real-world VA-ECMO practice because randomized trials have limited generalizability owing to highly qualified eligibility criteria, usually excluding patients who are expected to have futile outcomes due to ethical issues. [22, 24]

### AMI-CS vs. non-AMI-CS

In this study, patients with AMI-CS had lower in-hospital mortality rates than patients with non-AMI-CS. This association was consistently observed even after propensity score matching, suggesting that the observed difference was not solely attributable to confounding factors.

Adequate coronary revascularization facilitates myocardial recovery and improves clinical outcomes by addressing the underlying pathophysiological mechanism causing CS related to AMI [25, 26]. Accordingly, the culprit lesions of most patients with AMI-CS in this study were successfully revascularized. However, in patients with non-AMI-CS, identification of the causative factors of CS is often challenging owing to clinical heterogeneity [7, 8, 27]. Therefore, myocardial recovery may be delayed and insufficient in patients with non-AMI-CS. In particular, as nonischemic heart diseases, such as dilated cardiomyopathy, usually induce chronic cardiac remodeling and progressive impairment of the myocardial reserve [28], once CS develops in these patients, achieving sufficient myocardial recovery is often difficult unless long-term cardiac replacement therapy, such as HT or LVAD, is performed [5, 20]. Considering that the rate of cardiac replacement therapy has increased over time, further studies investigating the clinical outcomes of VA-ECMO in patients with non-AMI-CS are warranted.

In addition, this study identified several independent predictors of in-hospital mortality in patients with CS, which is consistent with the findings of previous studies [23, 29–31]. As mentioned, higher lactate levels are strongly correlated with increased mortality [23], as well as prolonged cardiac arrest duration [29]. The requirement for renal replacement therapy reflects the severity of the patient's condition and is associated with higher mortality [30]. Severe LV dysfunction has also been suggested as a predictor of poor clinical outcomes [31]. However, in our study, the LV ejection fraction was higher in patients who died within 24 h than in those who died after 24 h and survivors. This finding may be explained by the higher proportion of mixed or secondary shocks in this group, as shown in Supplemental Figure S3. In such cases, non-cardiac factors, such as systemic inflammation, sepsis, or multi-organ failure, may contribute to the overall shock state, masking the true severity of cardiac dysfunction [27]. In addition, previous studies mainly focused on patients with AMI-CS [31]. A recent multicenter retrospective trial focused on patients with non-AMI-CS and reported no significant association between LV ejection fraction and 30-day mortality but suggested a potential survival benefit of MCS use in patients with non-AMI-CS with severely reduced LV ejection fraction [32]. As the predictors for mortality in CS continue to be refined, additional studies focusing on the heterogeneity of non-AMI are needed to develop more accurate prognostic models.

### Study limitations

This study had several limitations. First, it has an inherent bias owing to its retrospective design. Our results should be interpreted as explanatory and hypothesis generating, given that causal relationships are not guaranteed owing to the nature of observational studies. Second, this study has limited generalizability because of the small sample size of the single-center registry. Further studies using multicenter data with larger patient populations are warranted. Third, the timing of LV ejection fraction measurement was not standardized, which may have introduced measurement bias and limited the comparability of LV ejection fraction values across patients. Fourth, the lack of additional serum markers, such as serum albumin level, limits our ability to comprehensively assess the nutritional or systemic status and its impact in this cohort. Finally, the effects of HT and durable MCS were not independently evaluated. Therefore, consecutive studies on long-term cardiac replacement strategies are required.

## Conclusions

In this single-center study, AMI-CS was associated with lower in-hospital mortality than non-AMI-CS after VA-ECMO treatment.

## Supplementary Information

Additional file 1.

## Data Availability

Data will not be shared due to the policy of the institutional review board.

## Abbreviations

AMI: Acute myocardial infarction
CS: Cardiogenic shock
ECMO: Extracorporeal membrane oxygenation
HF: Heart failure
HT: Heart transplantation
LVAD: Left ventricular assist device
MCS: Mechanical circulatory support
VA-ECMO: Venoarterial extracorporeal membrane oxygenation
