# Supplementary material for: Prognostic implication of venoarterial extracorporeal membrane oxygenation in acute myocardial infarction-related cardiogenic shock
Source: J Intensive Care. 2025 Jul 2;13:38. doi: 10.1186/s40560-025-00807-w (PMC12219146; doi:10.1186/s40560-025-00807-w)

**Supplemental Material**

***Prognostic Implication of Venoarterial Extracorporeal Membrane Oxygenation in Acute Myocardial Infarction-Related Cardiogenic Shock***

| Contents | Page |
| --- | --- |
| Supplemental Table S1. Lesion and procedural characteristics of patients with AMI-CS | 9 |
| Supplemental Table S2. Baseline characteristics stratified by initiation-to-death interval | 10 |
| Supplemental Table S3. Logistic regression models for in-hospital mortality after excluding patients who died within 24 h | 11 |
| Supplemental Table S4. Logistic regression models for in-hospital mortality after excluding patients who received ECPR | 11 |
| Supplemental Table S5. Baseline characteristics of the included patients before and after propensity score matching | 11 |
| Supplemental Table S6. Logistic regression models for in-hospital mortality after propensity score matching | 11 |
| Supplemental Figure S1. Distribution of CS etiologies | 9 |
| Supplemental Figure S2. Density plot of time-to-death data | 10 |
| Supplemental Figure S3. Distribution of CS etiologies stratified by the initiation-to-death interval | 10, 14 |
| Supplemental Figure S4. Trends in volume and running time of VA-ECMO | 11 |
| Supplemental Figure S5. Time-series plot of in-hospital mortality rates | 11 |

**Supplemental Table S1.** Lesion and procedural characteristics of patients with AMI-CSs

| **Characteristics** | **No. (%)** |  | **No. (%)** |
| --- | --- | --- | --- |
| **Type of MI**^a^ |  | **Total number of lesions treated** | 343 |
| ST-segment elevation MI | 176 (66.7%) | Stent | 281 (81.9%) |
| Non-ST-segment elevation MI | 49 (18.6%) | Balloon angioplasty | 41 (12.0%) |
| Type II MI | 17 (6.4%) | Thrombosuction alone | 6 (1.7%) |
| Type III-V MI | 22 (8.3%) | Guidewire crossing failure | 15 (4.4%) |
| **CAG done** | 255 (96.6%) | **Lesion stenosis (%)** | 92.9 ± 9.3 |
| **PCI done** | 216 (81.8%) | **Lesion ACC/AHA class** |  |
| **Access site** |  | B1 | 2 (0.6%) |
| Radial | 13 (5.2%) | B2 | 196 (57.1%) |
| Femoral | 239 (94.8%) | C | 145 (42.3%) |
| **Temporary pacemaker used** | 70 (27.8%) | **Initial TIMI grade** |  |
| **Number of diseased vessels** |  | Grade 0 | 153 (44.9%) |
| Single vessel disease | 42 (16.5%) | Grade 1 | 69 (20.2%) |
| Double vessel disease | 73 (28.7%) | Grade 2 | 119 (34.9%) |
| Triple or more disease | 126 (49.6%) | **Stent diameter (mm)** | 3.0 ± 0.4 |
| None or minimal | 13 (5.1%) | **Stent length (mm)** | 25.5 ± 8.3 |
| **Culprit vessel** |  | **Balloon used** |  |
| Left anterior descending artery | 104 (41.9%) | Pre-balloon | 196 (61.6%) |
| Left circumflex artery | 29 (11.7%) | Adjuvant balloon | 2 (0.6%) |
| Right coronary artery | 58 (23.4%) | Pre- and adjuvant balloon | 111 (34.9%) |
| Left main coronary artery | 43 (17.3%) | None | 9 (2.8%) |
| Multi-vessel | 11 (4.4%) | **Number of stents per patient** | 1.3 ± 0.9 |
| Graft vessel | 3 (1.2%) | **Procedural success** | 170 (78.7%) |

Data are presented as frequency (%) or mean ± standard deviation. Procedural success was defined as TIMI grade III flow recovery in the culprit artery.

ACC/AHA, American College of Cardiology/American Heart Association; AMI-CS, acute myocardial infarction cardiogenic shock; CAG, coronary angiography; MI, myocardial infarction; PCI, percutaneous coronary intervention; TIMI, Thrombolysis In MI.
^a^ The type of myocardial infarction was defined according to the 4^th^ Universal Definition of MI.

**Supplemental Table S2.** Baseline characteristics stratified by initiation-to-death interval

| **Variables** | **Initiation-to-death interval** | | |  |  |
| --- | --- | --- | --- | --- | --- |
|  | **<24 h**  **(N=145)** | **≥24 h**  **(N=297)** | **In-hospital death**  **(N=442)** | **Survivor**  **(N=225)** | ***P*-value**^a^ |
| AMI-CS patients | 51 (35.2) | 108 (36.4) | 159 (36.0) | 105 (46.7) | 0.027 |
| Age, years | 70 (57–78) | 65 (53–73) | 66 (54–75) | 59 (47–67) | <0.001 |
| Male | 90 (62.1) | 202 (68.0) | 292 (66.1) | 157 (69.8) | 0.287 |
| ECPR | 95 (65.5) | 119 (40.1) | 214 (48.4) | 52 (23.1) | <0.001 |
| Cardiac arrest | 116 (80.0) | 176 (59.3) | 292 (66.1) | 117 (52.0) | <0.001 |
| CPR time (min) | 36 (20–52) | 26 (17–46) | 29 (18–50) | 17 (7–34) | <0.001 |
| CPR rhythm |  |  |  |  | <0.001 |
| Shockable | 15 (12.9) | 53 (30.1) | 68 (23.3) | 55 (47.0) |  |
| Non-shockable | 92 (79.3) | 107 (60.8) | 199 (68.3) | 48 (41.0) |  |
| Unknown | 9 (7.8) | 16 (9.1) | 25 (8.6) | 14 (12.0) |  |
| LV ejection fraction (%) | 46 (26–62) | 19 (12–34) | 22 (14–46) | 23 (16–37) | <0.001 |
| SCAI-CSWG shock stage |  |  |  |  | <0.001 |
| Stage C | 0 (0.0) | 13 (4.4) | 13 (2.9) | 27 (12.0) |  |
| Stage D | 3 (2.1) | 37 (12.5) | 40 (9.0) | 46 (20.4) |  |
| Stage E | 142 (97.9) | 247 (83.2) | 389 (88.0) | 152 (67.6) |  |
| Renal replacement therapy | 59 (40.7) | 233 (78.7) | 292 (66.2) | 80 (35.6) | <0.001 |
| LV unloading | 0 (0.0) | 22 (7.4) | 22 (5.0) | 13 (5.8) | 0.004 |
| HT or LVAD | 0 (0.0) | 20 (6.7) | 20 (4.5) | 43 (19.1) | <0.001 |
| Hemoglobin, g/dL | 9.8 (8.1–12.5) | 10.9 (8.6–12.9) | 10.6 (8.5–12.9) | 12.2 (10.1–14.1) | <0.001 |
| Platelet count, /μL | 145,000  (73,000–205,000) | 152,000  (81,000–227,000) | 149,500  (80,000–222,000) | 179,000  (129,000–246,000) | <0.001 |
| AST, IU/L | 124 (34–414) | 99 (36–331) | 100 (35–377) | 78 (33–216) | 0.097 |
| ALT, IU/L | 63 (24–226) | 63 (25–224) | 63 (24–225) | 59 (27–184) | 0.890 |
| Creatinine, mg/dL | 1.23 (0.96–1.94) | 1.44 (1.08–2.06) | 1.38 (1.04–2.00) | 1.24 (0.99–1.62) | 0.001 |
| pH | 7.14 (7.01–7.28) | 7.19 (7.07–7.28) | 7.18 (7.04–7.28) | 7.27 (7.17–7.37) | <0.001 |
| Lactate |  |  |  |  |  |
| Value, mmol/L | 14.0 (7.4–18.5) | 12.2 (5.9–18.2) | 12.7 (6.8–18.4) | 6.3 (3.3–10.8) | <0.001 |
| ≥8.0 mmol/L | 89 (71.8) | 175 (69.2) | 264 (70.0) | 79 (38.9) | <0.001 |
| Length of hospital stay, days | 0 (0–1) | 9 (2–19) | 3 (1–12) | 32 (19–63) | <0.001 |

Data are presented as medians (interquartile ranges) or frequencies (%). AMI-CS, acute myocardial infarction-cardiogenic shock; ALT, alanine aminotransferase; AST, aspartate aminotransferase; CPR, cardiopulmonary resuscitation; ECPR, extracorporeal cardiopulmonary resuscitation; HT, heart transplantation; IU, international unit; LV, left ventricle; LVAD, left ventricle assist device; SCAI-CSWG, Society for Cardiovascular Angiography and Interventions and Cardiogenic Shock Working Group
^a^ *P*-value represents comparison among patients who died within 24 h after initiation, those died after 24 h, and survivors.

**Supplemental Table S3.** Logistic regression models for in-hospital mortality after excluding patients who died within 24 h

| **Variables** | **Univariate model** | | **Multivariate model** | |
| --- | --- | --- | --- | --- |
|  | **OR (95% CI)** | ***P*-value** | **OR (95% CI)** | ***P*-value** |
| AMI-CS patients | 0.63 (0.43–0.92) | 0.017 | 0.50 (0.30–0.85) | 0.011 |
| Age | 1.02 (1.01–1.03) | 0.003 | 1.03 (1.01–1.04) | 0.003 |
| Male | 0.85 (0.58–1.26) | 0.433 | 1.20 (0.73–2.00) | 0.474 |
| Prolonged cardiac arrest^a^ | 1.96 (1.24–3.13) | 0.004 | 1.99 (1.12–3.58) | 0.021 |
| Severe LV dysfunction^b^ | 1.54 (1.04–2.29) | 0.033 | 1.72 (1.04–2.88) | 0.037 |
| Renal replacement therapy | 6.63 (4.39–10.16) | <0.001 | 5.06 (3.16–8.20) | <0.001 |
| LV unloading | 1.36 (0.68–2.85) | 0.394 | - | - |
| Hemoglobin | 0.84 (0.78–0.90) | <0.001 | 0.89 (0.81–0.97) | 0.013 |
| Platelet count per 1,000/μL | 0.06 (0.01–0.42) | 0.005 | 0.31 (0.03–3.61) | 0.348 |
| AST per 100 IU/L | 1.02 (1.00–1.04) | 0.029 | 1.02 (0.98–1.07) | 0.309 |
| ALT per 100 IU/L | 1.03 (1.00–1.06) | 0.056 | 0.97 (0.91–1.04) | 0.403 |
| Creatinine | 1.30 (1.11–1.57) | 0.003 | 0.98 (0.81–1.19) | 0.813 |
| pH<7.2 | 2.06 (1.41–3.03) | <0.001 | 1.32 (0.79–2.20) | 0.289 |
| Lactate ≥8.0 mmol/L | 3.69 (2.51–5.46) | <0.001 | 2.77 (1.67–4.65) | <0.001 |

AMI-CS, acute myocardial infarction-cardiogenic shock; ALT, alanine aminotransferase; AST, aspartate aminotransferase; CI, confidence interval; IU, international unit; LV, left ventricle; OR, odds ratio.
^a^ Prolonged cardiac arrest indicates an arrest of duration >30 min.
^b^ Severe LV dysfunction indicates an LV ejection fraction <30%.

**Supplemental Table S4.** Logistic regression models for in-hospital mortality after excluding patients who received ECPR

| **Variables** | **Univariate model** | | **Multivariate model** | |
| --- | --- | --- | --- | --- |
|  | **OR (95% CI)** | ***P*-value** | **OR (95% CI)** | ***P*-value** |
| AMI-CS patients | 0.64 (0.41–1.01) | 0.055 | 0.49 (0.26–0.91) | 0.025 |
| Age | 1.03 (1.01–1.04) | <0.001 | 1.03 (1.01–1.05) | 0.001 |
| Male | 0.92 (0.59–1.43) | 0.707 | 1.21 (0.69–2.13) | 0.501 |
| Prolonged cardiac arrest^a^ | 1.34 (0.60–3.14) | 0.489 | 1.52 (0.59–4.08) | 0.391 |
| Severe LV dysfunction^b^ | 1.26 (0.81–1.98) | 0.305 | 1.75 (0.99–3.13) | 0.056 |
| Renal replacement therapy | 4.76 (3.01–7.65) | <0.001 | 3.76 (2.20–6.49) | <0.001 |
| LV unloading | 0.65 (0.26–1.62) | 0.356 | - | - |
| Hemoglobin | 0.84 (0.78–0.91) | <0.001 | 0.91 (0.82–1.01) | 0.083 |
| Platelet count per 1,000/μL | 0.03 (0.00–0.30) | 0.003 | 0.14 (0.01–1.94) | 0.143 |
| AST per 100 IU/L | 1.02 (1.00–1.04) | 0.061 | 1.01 (0.98–1.04) | 0.443 |
| ALT per 100 IU/L | 1.02 (1.00–1.05) | 0.011 | 0.99 (0.95–1.04) | 0.661 |
| Creatinine | 1.36 (1.10–1.73) | 0.009 | 1.00 (0.79–1.30) | 0.980 |
| pH<7.2 | 2.16 (1.38–3.41) | 0.001 | 1.79 (0.99–3.26) | 0.053 |
| Lactate ≥8.0 mmol/L | 3.61 (2.31–5.70) | <0.001 | 2.33 (1.31–4.17) | 0.004 |

AMI-CS, acute myocardial infarction-cardiogenic shock; ALT, alanine aminotransferase; AST, aspartate aminotransferase; CI, confidence interval; ECPR; extracorporeal cardiopulmonary resuscitation; IU, international unit; LV, left ventricle; OR, odds ratio.
^a^ Prolonged cardiac arrest indicates an arrest of duration >30 min.
^b^ Severe LV dysfunction indicates an LV ejection fraction <30%.

**Supplemental Table S5.** Baseline characteristics of the included patients before and after propensity score matching

| **Variables** | **Before Propensity Score Matching** | | ***P*-value** | **After Propensity Score Matching** | | ***P*-value** | |
| --- | --- | --- | --- | --- | --- | --- | --- |
|  | **AMI-CS group**  **(N=264)** | **Non-AMI-CS group**  **(N=403)** |  | **AMI-CS group**  **(N=147)** | **Non-AMI-CS group**  **(N=147)** |  |  |
| Age, years | 66 (58–74) | 61 (45–72) | <0.001 | 65 (54–73) | 67 (55–77) | 0.216 |  |
| Male | 218 (82.6) | 231 (57.3) | <0.001 | 110 (74.8) | 110 (74.8) | 1.000 |  |
| ECPR | 119 (45.1) | 147 (36.5) | 0.033 | 68 (46.3) | 63 (42.9) | 0.639 |  |
| Cardiac arrest | 184 (69.7) | 225 (55.8) | <0.001 | 104 (70.7) | 96 (65.3) | 0.381 |  |
| CPR time (min) | 24 (14–44) | 28 (15–43) | 0.369 | 26 (14–45) | 24 (12–41) | 0.451 |  |
| CPR rhythm |  |  | <0.001 |  |  | 0.118 |  |
| Shockable | 75 (40.8) | 48 (21.3) |  | 40 (38.5) | 24 (25.0) |  |  |
| Non-shockable | 89 (48.4) | 158 (70.2) |  | 51 (49.0) | 59 (61.5) |  |  |
| Unknown | 20 (10.9) | 19 (8.4) |  | 13 (12.5) | 13 (13.5) |  |  |
| LV ejection fraction (%) | 20 (13–31) | 25 (15–52) | <0.001 | 21 (15–32) | 19 (14–36) | 0.809 |  |
| SCAI-CSWG shock stage |  |  | 0.091 |  |  | 0.599 |  |
| Stage C | 15 (5.7) | 25 (6.2) |  | 9 (6.1) | 9 (6.1) |  |  |
| Stage D | 25 (9.5) | 61 (15.1) |  | 11 (7.5) | 16 (10.9) |  |  |
| Stage E | 224 (84.9) | 317 (78.7) |  | 127 (86.4) | 122 (83.0) |  |  |
| Renal replacement therapy | 131 (49.6) | 241 (60.0) | 0.011 | 85 (57.8) | 88 (59.9) | 0.813 |  |
| LV unloading | 6 (2.3) | 29 (7.2) | 0.009 | 6 (4.1) | 7 (4.8) | 1.000 |  |
| HT or LVAD | 7 (2.7) | 56 (13.9) | <0.001 | 6 (4.1) | 21 (14.3) | 0.005 |  |
| Hemoglobin, g/dL | 12.1 (10.1–14.4) | 10.2 (8.6–12.6) | <0.001 | 11.6 (9.1–13.8) | 11.3 (9.2–13.2) | 0.632 |  |
| Platelet count, /μL | 193,000  (140,000–255,500) | 135,000  (76,000–203,000) | <0.001 | 169,000  (114,000–232,500) | 158,000  (96,500–243,500) | 0.634 |  |
| AST, IU/L | 82 (33–280) | 101 (36–302) | 0.213 | 90 (37–331) | 96 (34–238) | 0.500 |  |
| ALT, IU/L | 60 (27–167) | 65 (25–243) | 0.483 | 74 (34–213) | 60 (25–183) | 0.164 |  |
| Creatinine, mg/dL | 1.33 (1.06–1.71) | 1.34 (1.00–2.01) | 0.845 | 1.35 (1.06–1.86) | 1.41 (1.06–2.12) | 0.379 |  |
| pH | 7.20 (7.06–7.30) | 7.22 (7.10–7.33) | 0.020 | 7.21 (7.04–7.31) | 7.23 (7.10–7.33) | 0.230 |  |
| Lactate |  |  |  |  |  |  |  |
| Value, mmol/L | 10.6 (5.8–15.1) | 10.1 (4.2–17.3) | 0.535 | 11.3 (5.6–15.9) | 10.1 (5.0–16.8) | 0.511 |  |
| ≥8.0 mmol/L | 145 (63.9) | 198 (56.1) | 0.076 | 95 (64.6) | 83 (56.8) | 0.214 |  |
| Length of hospital stay, days | 12 (2–26) | 10 (1–33) | 0.645 | 13 (3–31) | 9 (1–31) | 0.164 |  |

Data are presented as the median (interquartile range) or number (%). AMI-CS, acute myocardial infarction-cardiogenic shock; ALT, alanine aminotransferase; AST, aspartate aminotransferase; CPR, cardiopulmonary resuscitation; ECPR, extracorporeal cardiopulmonary resuscitation; HT, heart transplantation; IU, international unit; LV, left ventricle; LVAD, left ventricle assist device; SCAI-CSWG, Society for Cardiovascular Angiography and Interventions and Cardiogenic Shock Working Group

**Supplemental Table S6.** Logistic regression models for in-hospital mortality after propensity score matching

| **Variables** | **Univariate model** | | **Multivariate model** | |
| --- | --- | --- | --- | --- |
|  | **OR (95% CI)** | ***P*-value** | **OR (95% CI)** | ***P*-value** |
| AMI-CS patients | 0.53 (0.32–0.86) | 0.011 | 0.33 (0.18–0.62) | <0.001 |
| Age | 1.05 (1.03–1.07) | <0.001 | 1.07 (1.04–1.10) | <0.001 |
| Male | 0.79 (0.44–1.37) | 0.411 | 0.98 (0.47–2.00) | 0.954 |
| Prolonged cardiac arrest^a^ | 2.22 (1.25–4.10) | 0.008 | 3.65 (1.74–8.08) | <0.001 |
| Severe LV dysfunction^b^ | 1.65 (0.97–2.77) | 0.061 | 2.49 (1.22–5.20) | 0.013 |
| Renal replacement therapy | 2.62 (1.61–4.31) | <0.001 | 2.47 (1.31–4.75) | 0.006 |
| Left ventricle unloading | 0.86 (0.28–2.9) | 0.791 | - | - |
| Hemoglobin | 0.84 (0.77–0.92) | <0.001 | 0.89 (0.78–1.00) | 0.049 |
| Platelet count per 1,000/μL | 0.21 (0.02–2.29) | 0.201 | 0.54 (0.03–13.36) | 0.698 |
| AST per 100 IU/L | 1.01 (1.00–1.03) | 0.268 | 1.01 (0.96–1.09) | 0.665 |
| ALT per 100 IU/L | 1.01 (0.98–1.04) | 0.545 | 0.98 (0.87–1.08) | 0.732 |
| Creatinine | 1.41 (1.11–1.87) | 0.010 | 1.21 (0.89–1.71) | 0.261 |
| pH<7.2 | 3.18 (1.92–5.39) | <0.001 | 2.13 (1.07–4.29) | 0.032 |
| Lactate ≥8.0 mmol/L | 3.94 (2.39–6.58) | <0.001 | 3.79 (1.92–7.68) | <0.001 |

AMI-CS, acute myocardial infarction-cardiogenic shock; ALT, alanine aminotransferase; AST, aspartate aminotransferase; CI, confidence interval; IU, international unit; LV, left ventricle; OR, odds ratio.
^a^ Prolonged cardiac arrest indicates an arrest of duration >30 min.
^b^ Severe LV dysfunction indicates an LV ejection fraction <30%.

**Supplemental Figure S1.** Distribution of CS etiologies

The pie chart represents the distribution of patients based on the etiology of CS. The percentages indicate the proportion of the total population. Abbreviations: AMI, acute myocardial infarction; CS, cardiogenic shock; HF, heart failure.


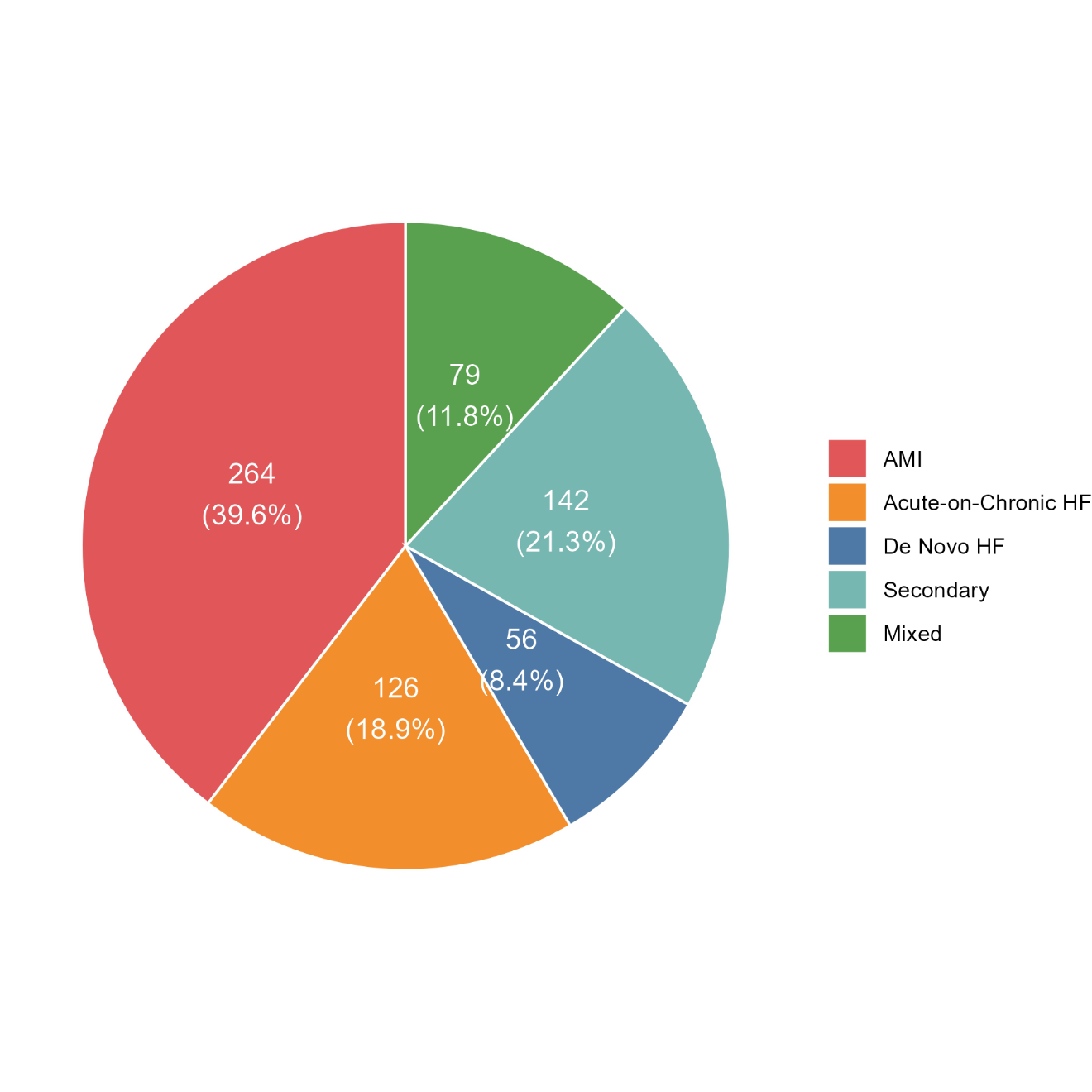


**Supplemental Figure S2.** Density plot of time-to-death data

Density plot showing the distribution of time-to-death intervals in patients treated with VA-ECMO. Mortality peaked at approximately 24 h after VA-ECMO initiation (first dashed line) and then decreased, reaching a plateau approximately 7-days later (second dashed line). Abbreviations: VA-ECMO, venoarterial extracorporeal membrane oxygenation.


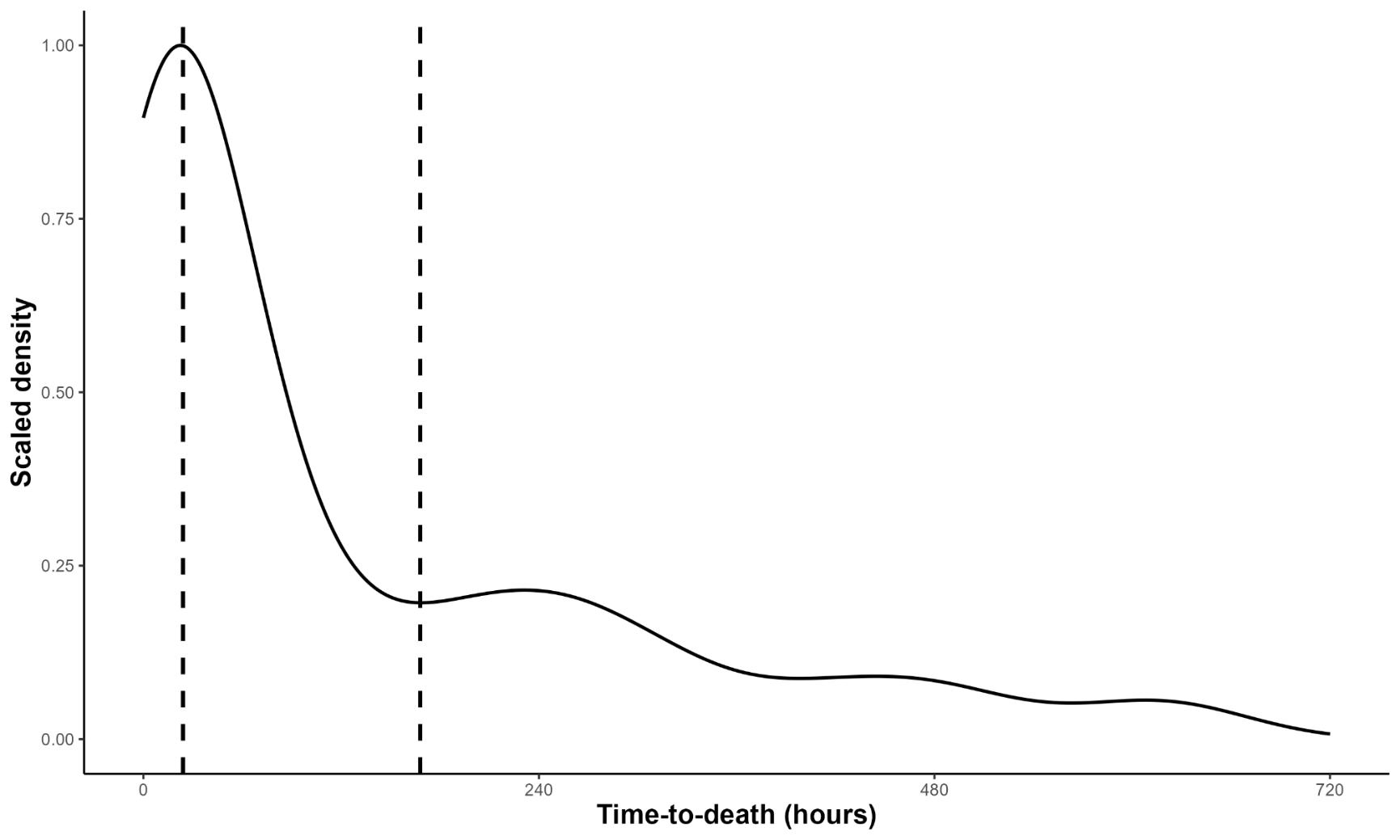


**Supplemental Figure S3.** Distribution of CS etiologies stratified by the initiation-to-death interval

Stacked bar plot of the distribution of CS etiologies according to the initiation-to-death interval. The intervals include patients who died within 24 h (<24 h), those who died after 24 h (≥24 h), and survivors. Abbreviations: AMI, acute myocardial infarction; CS, cardiogenic shock; HF, heart failure.


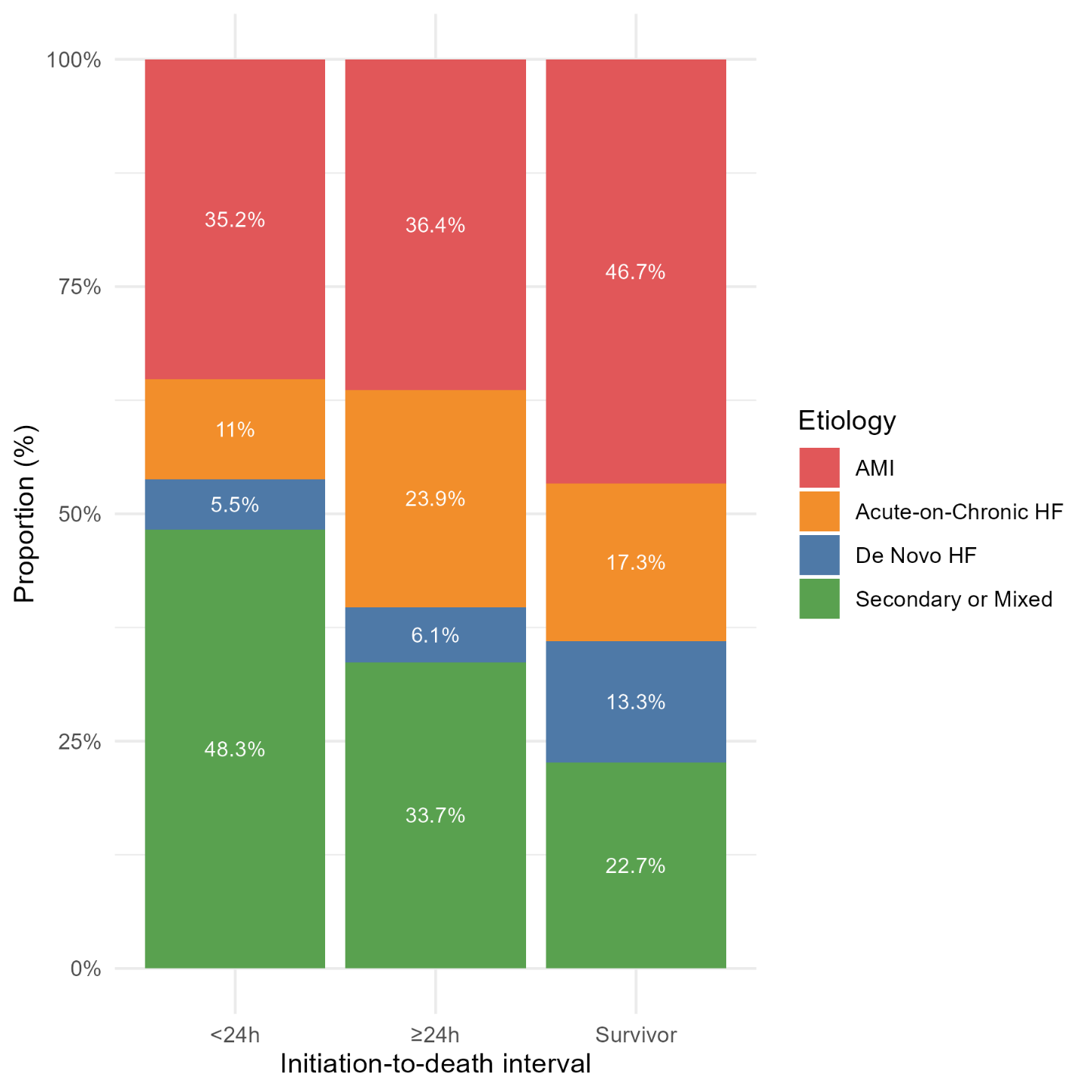


**Supplemental Figure S4.** Trends in volume and running time of VA-ECMO

The bar chart represents the annual volume of VA-ECMO cases, and the line chart shows the median VA-ECMO running time (h) for each year. A significant increasing trend is observed in both the VA-ECMO volume and running time over the years (*P* for trend <0.001). *P*-values were calculated using linear regression. Abbreviations: VA-ECMO, venoarterial extracorporeal membrane oxygenation.


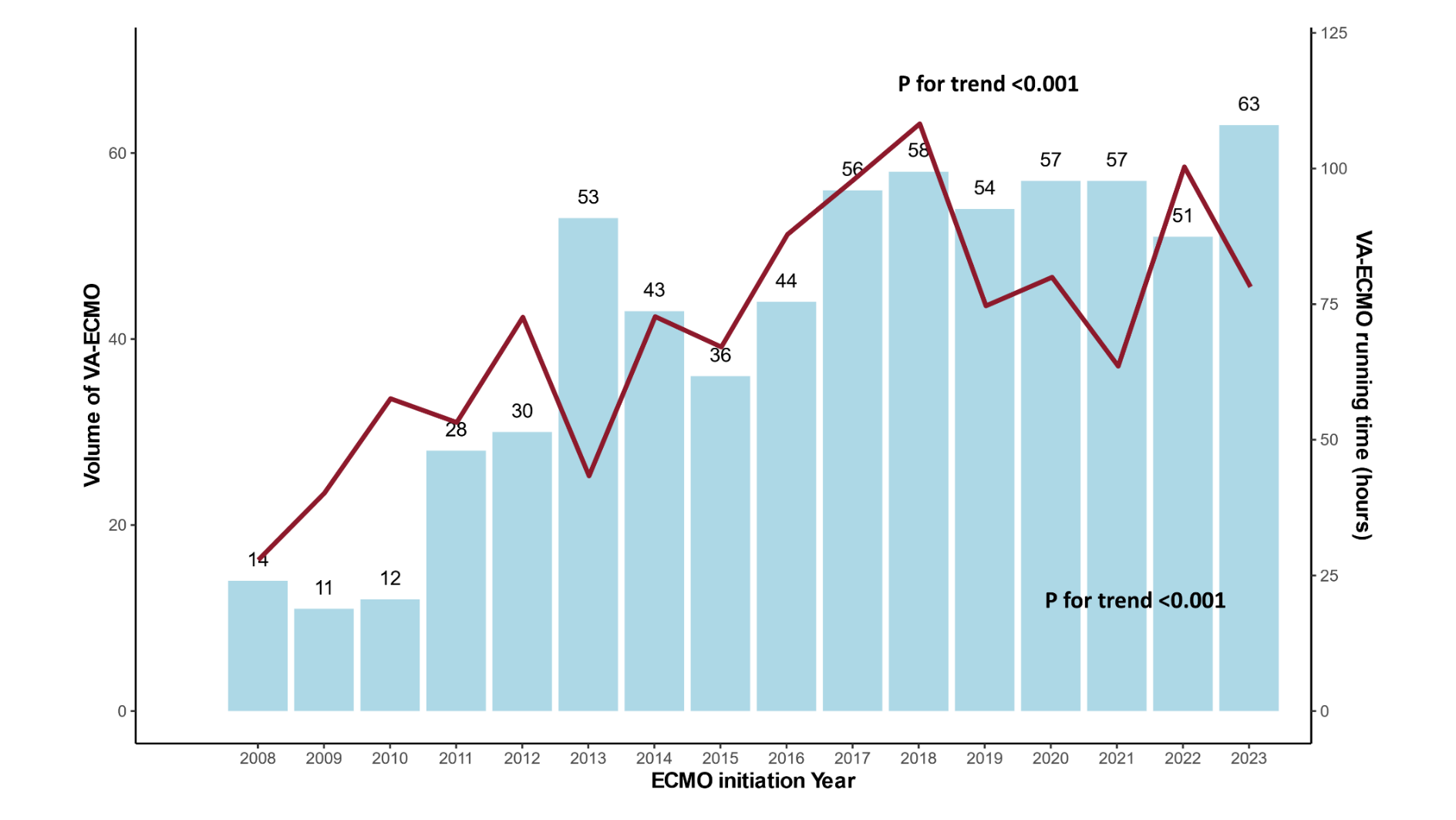


**Supplemental Figure S5.** Time-series plot of in-hospital mortality rates

In-hospital mortality rates over time in the overall study population (**A**), AMI-CS group (**B**), and non-AMI-CS group (**C**). The *P*-value for the trend indicates the significance of the trend and was calculated using linear regression analysis. Abbreviations: AMI, acute myocardial infarction; CS, cardiogenic shock; ECMO, extracorporeal membrane oxygenation.


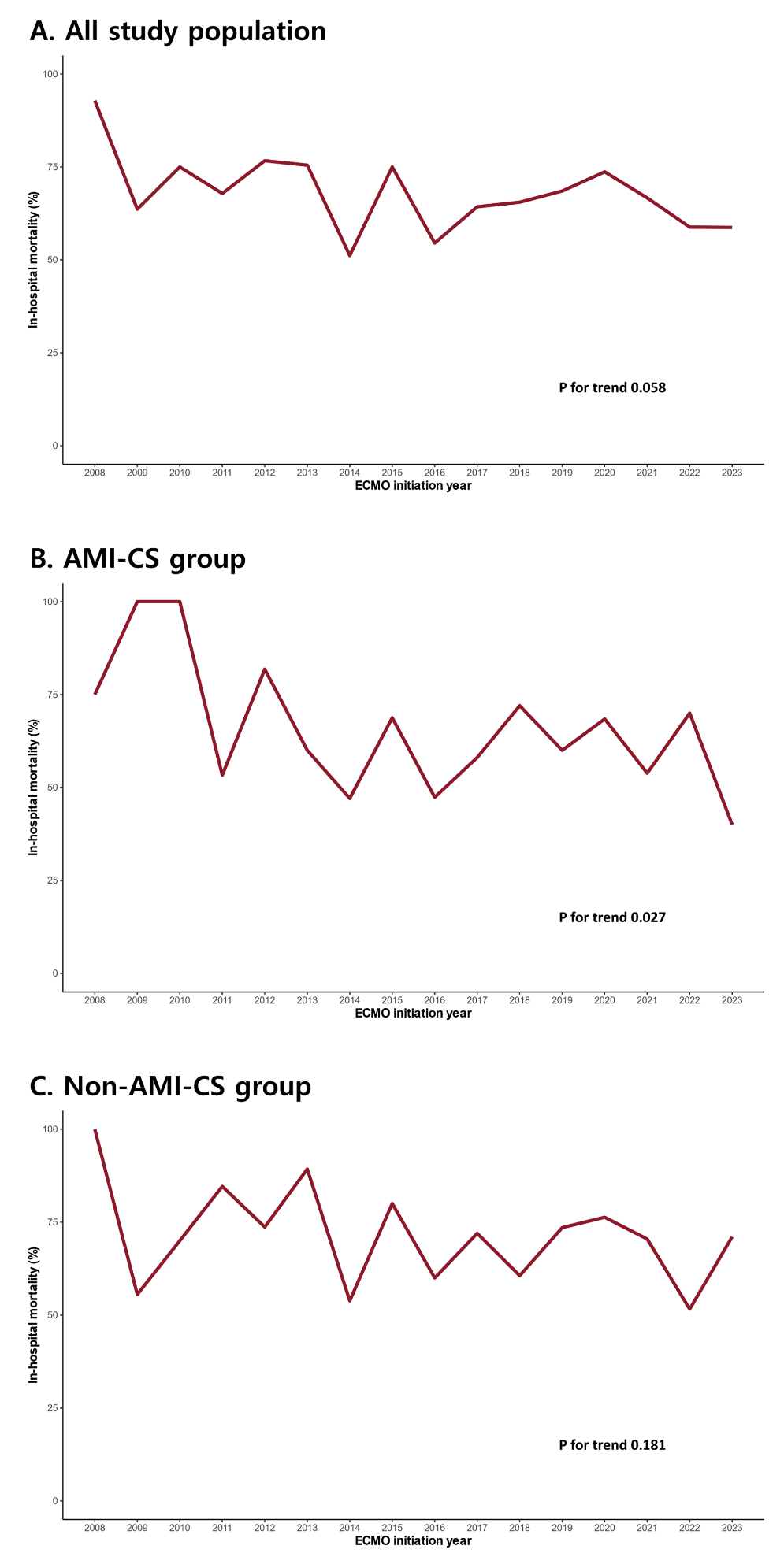

Supplement: Supplementary file 1 — Additional file 1. [file 40560_2025_807_MOESM1_ESM.docx]
